# Quality and safety attributes of afghan raisins before and after processing

**DOI:** 10.1002/fsn3.190

**Published:** 2014-12-15

**Authors:** Stacy McCoy, Jun Won Chang, Kevin T McNamara, Haley F Oliver, Amanda J Deering

**Affiliations:** 1 Department of Agricultural Economics, Purdue UniversityWest Lafayette, Indiana; 2 Department of Botany and Plant Pathology, Purdue UniversityWest Lafayette, Indiana; 3 Department of Food Science, Purdue UniversityWest Lafayette, Indiana

**Keywords:** Afghanistan, quality, raisin, safety

## Abstract

Raisins are an important export commodity for Afghanistan; however, Afghan packers are unable to export to markets seeking high-quality products due to limited knowledge regarding their quality and safety. To evaluate this, Afghan raisin samples from pre-, semi-, and postprocessed raisins were obtained from a raisin packer in Kabul, Afghanistan. The raisins were analyzed and compared to U.S. standards for processed raisins. The samples tested did not meet U.S. industry standards for embedded sand and pieces of stem, total soluble solids, and titratable acidity. The Afghan raisins did meet or exceed U.S. Grade A standard for the number of cap-stems, percent damaged, crystallization levels, moisture content, and color. Following processing, the number of total aerobic bacteria, yeasts, molds, and total coliforms were within the acceptable limits. Although quality issues are present in the Afghan raisins, the process used to clean the raisins is suitable to maintain food safety standards.

## Introduction

Raisins are Afghanistan's top agricultural export, contributing 17% of all licit agricultural export value (Jalal 2012). Afghanistan's high evaporation rate (low rainfall/humidity combined with high temperature) creates the world's most favorable climate for raisin production, followed closely by Iran and California (Coombe and Dry 1992). Afghanistan is forecasted to produce 33,000 metric tons (MT) of raisins in the 2013/14 marketing year (MY; October to September), which is 2.75% of the 1.2 million MT global supply (Bunnel and Safi 2013; Halstead 2013).

Afghan sun-dried, dark raisins (*aftabi*) are dried in direct sunlight and receive lower prices than shade-dried, green raisins (*kishmish*). Thus, growers dry sun-dried raisins on the ground (or any other available space) are produced from “trimming waste, shattered berries, wasp-damaged, spillage, and left-over fruit” that could not be shade-dried (Lister et al. 2004).

Several factors impact Afghanistan's MY 13/14 raisin forecast, which is a 1.5% increase from MY 2012/13 (Bunnel and Safi 2013). These factors include the planting of new vines in 2010 and improved efficiency of irrigation water. However, fungal infections of powdery mildew, botrytis, and anthracnose affected vineyards in Kandahar and Zabul provinces, reducing grape quality at harvest (Bunnel and Safi 2013). Consumers may notice a diminished quality in both grape and raisin products, though reduced quality will be less noticeable in raisins.

Afghan growers typically directly sell their own raisins at local wholesale markets. The median landholding of an Afghan who grows grapes, often in a vineyards or *bagh-i-angur* surrounded by mud walls, is one jerib or 0.5 acre (0.2 ha) (Afghanistan Government of the Islamic Republic 2014). Intermediaries purchase raisins from hundreds of growers, and then sell them to raisin packers who ultimately export raisins (Lister et al. 2004). Afghanistan's largest raisin exporter, Tabasom, sources raisins from ∼3000 growers (Oliver 2012). The intermediary step creates a separation from raisin grower to raisin packer, which means raisin packers have little direct communication with growers and exert limited control over raw material quality. Most importantly, growers do not receive a price incentive for production of a higher quality product (Lister et al. 2004).

Horticultural studies from the U.S. dating back to the 1920s clarify how growing, harvest, drying, and storage factors impact the quality of raisins, thus guiding growers and packers how to produce high quality raisins for the U.S. and European markets (Chace and Church 1927; Jacob 1944; Christensen et al. 1995; Doymaz and Pala 2002; Angulo et al. 2007). Since the fall of the Taliban in late 2001, numerous case studies have documented the poor harvesting and handling practices that inhibit Afghans from producing higher quality raisins, and thus limit the growth of the export market (Chehrezad and Fite 2005; Chubb et al. 2005; U.S. Agency for International Development 2011). However, a 1991 study found Afghan raisins imported into Europe had comparable quality levels to other raisins on the European retail market for six attributes (Bongers et al. 1991).

Importantly, no known study has objectively measured the food safety levels of Afghan raisins. Food safety is critical to Afghanistan's export potential to countries with high food safety standards; Afghanistan packers have had 13 raisin shipments rejected at EU external borders since 2002 for molds or mycotoxins, compared to 12 rejections of raisins from Turkey, the world's largest raisin exporter (Rapid Alert System for Food and Feed (RASFF) 2014);. However, Turkey exports ∼145 times more raisins to the EU (1,860,055 vs. 12,678 MT) compared to Afghanistan over the same time period (United Nations Food and Agriculture Organization 2013). Afghan raisin packers are motivated to improve raisin quality so they can export to markets seeking higher quality product, such as the U.S. and EU (Osmani, 2012, pers. comm.). However, the minimal analysis and availability of data for Afghan raisin safety and quality levels hinder the ability of Afghan raisin packers to choose which factors they should focus their efforts to improve raisin quality. In this study, the quality and safety aspects of pre-, semi-, and postprocessed Afghan raisins were evaluated from multiple production lots in order to partially fill this data gap.

## Materials and Methods

### Raisin sample source and characteristics

Tabasom, a premier raisin packer in Kabul, Afghanistan, provided 15 samples (2 kg each) of sun dried (*aftabi)* raisins (Thompson Seedless variety) to evaluate quality and safety standards. Raisins processed in the Tabasom factory are the highest quality raisins currently available in Afghanistan and are responsible for 60% of all Afghan raisin exports (Osmani, 2012, pers. comm.). There are only 8 known raisin processors in Afghanistan, with only Tabasom using laser sorters. The sample size was limited by (a) Cost preventing shipment of hundreds of samples to the U.S., and (b) the goal to see if the best quality raisins coming from Afghanistan meet U.S. standards. Nine samples were fully processed raisins, three samples were semiprocessed and three samples were unprocessed raisins. The “fully” processed samples were considered to be ready for export, whereas the “unprocessed” samples were direct from grower fields. The “semi” processed samples were taken during midprocessing after one washing. Tabasom processes raisins by double-washing the raisins to remove embedded dirt and debris, then sorts them by size, and uses an X-ray machine to identify and remove foreign material including any stems remaining from the washing stage (Bunnel and Safi 2013). Samples were brought to the U.S. and subsequently shipped to Purdue University, West Lafayette, IN, and analyzed in the Department of Food Science. The sample processing followed inspection methodologies from the USDA or the FDA, or methodologies from the previous raisin studies (Bongers et al. 1991; Petrucci and Clary 2002; Angulo et al. 2007). As standards values are set for raisins ready for human consumption, the focus of this study was on the fully processed raisins.

### Embedded sand in processed raisins

Five 100 g samples of raisins were boiled in water for approximately 5 min. The raisins were removed and the water drained through filter paper. Once the paper dried, it was weighed to measure the residual amount of sand/dirt.

### Crystallization qualification and quantification

Crystallized raisins were visually identified by comparing individual raisins from a 16 oz (∼450 g) sample to textbook photos (Petrucci and Clary 2002). These raisins were set aside, and weighed after the entire sample was inspected. This visual inspection protocol was also used for inspecting cap-stems, damage, and discoloration.

### Quantification of cap-stems and pieces of stems

Pieces of stem were visually identified and counted for an entire 2 kg sample. As each Afghan raisin sample was only 2 kg and the U.S. standard is for a 2.72 kg sample, the results were multiplied by 1.36 for each sample for comparison to the U.S. standard (U.S. Department of Agriculture 1978).

### Damage and moisture content assessment

Five of the nine processed raisin samples were examined for damage, defined as “raisins affected by sunburn, scars, insect injury, mechanical injury, or other similar means which seriously affect the appearance, edibility, storage quality, or shipping quality of the raisins” (U.S. Department of Agriculture 1978). As the study had 3 samples each of pre- and semiprocessed raisins, for comparison only a random selection of 5 unprocessed samples were tested. A 100 g sample from 5 of the 9 processed raisin samples, and from all 3 of the semiprocessed and unprocessed raisin samples were individually blended (Oster® blender, Rye, NY) until a homogenous mixture was obtained. A portion of the 100 g sample was used to measure the water activity for each sample following the manufacturer's directions (Aqua Lab 4TE Water Activity Meter, Pullman, WA) and the resulting values averaged across the pre-, semi-, and postprocessed samples.

### Discoloration assessment

A colorimeter (HunterLab Inc. LabScan XE 16305; HunterLab Universal Software ver. 3.71, Reston, VA) was used to collect color data from the Afghan raisins to compare color values of other raisins reported in the viticulture literature (Angulo et al. 2007). Colorimetric measurements were obtained from 10 raisins from 5 of the 9 processed raisin samples, and 10 raisins from all 3 of the semiprocessed and unprocessed raisin samples.

### Total soluble solids

To determine soluble solids, 25 g of raisins and 100 mL distilled water were blended (Oster® blender) for 3 min at a low speed and the samples were strained through cheesecloth to remove remaining particles (Bongers et al. 1991; WANG 2014). The sample was placed on a refractometer (Maselli Misure, Stockton, CA), 3 readings for each of the 9 processed samples were obtained, and the overall average was calculated. Other studies comparing Brix values to final raisin quality (as measured by an air stream sorter) had access to the fresh grapes used for the dried raisins. This study did not have access to the grapes from which the raisins were produced, thus used the methodology in Bongers et al., which measured Brix of raisins. Brix measures total soluble solids (TSS, and not a percentage); thus the dilution of the raisins does not bias the resulting measurement.

### Titratable acidity

Using the same suspension from the TSS test, the TA was measured by titrating 25 mL of filtrate with a 0.1N sodium hydroxide solution to the phenolphthalein end-point and expressed as percent anhydrous tartaric acid. The % dry weight as 
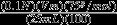
 was then calculated (Bongers et al. 1991; Wang 2014).

### Raisin grading by size

USDA grades raisins into select, small (midget), and mixed sizes (neither select nor small) based on the percentage of a sample that passes through screens with specified hole sizes (U.S. Department of Agriculture 1978). For select size raisins, no more than 60% by weight of all the raisins will pass through holes 22/64 in. in diameter and not more than 10% may pass through holes 20/64 in. in diameter. For small raisins, 95% will pass through holes 24/64 in. in diameter and not less than 70% will pass through holes 22/64 in. in diameter. A total of 500 g from each of five processed raisin samples were passed through a series of three screens corresponding to USDA sizes. The raisins that passed through each screen were then weighed.

### Microbial assessment of raisins

#### Total aerobic plate count

Sampling and plating methods were based on the Bacteriological Analytical Manual (BAM) by FDA for Aerobic Plate Count (Maturin and Peeler 2001). Five 25 g raisin samples were taken from each of the processed, semiprocessed, and unprocessed raisin samples. Each sample was blended in 225 mL of 0.1 mol/L phosphate buffer, pH 7.0 (PB) for 2 min (Oster® blender). Serial dilutions were performed using PB as the diluent and the samples were spread-plated on Plate Count Agar (BD, Sparks, MD). The plates were incubated for 48 h at 30°C and the average CFU/g of total aerobic bacteria was calculated.

#### Yeast and mold quantification

All methods used were based on the BAM by the U.S. FDA for enumeration of yeasts and molds in food (Tournas et al. 2001). Five 25 g raisin samples from each of the processed, semiprocessed, and unprocessed raisin samples were added to sterile blender bottles with 225 mL of PB. The samples were blended for 2 min to obtain a homogeneous mixture (Oster® blender). The samples were then serially diluted in PB and spread-plated on Potato Dextrose Agar (PDA; BD), Rose-bengal Agar (RB; Hardy Diagnostics, Santa Maria, CA), and Corn Meal Agar (CM; BD). The plates were incubated at 25°C for 5 days and the number of yeasts and molds enumerated and expressed as CFU/g.

#### Quantification of total coliforms

Total coliforms were enumerated from the raisin samples using 3M Petrifilm™ Coliform Count (CC; 3M Microbiology Product, St. Paul, MN). Using sampling methods from the BAM for enumeration of *Escherichia coli* and coliforms from food (Feng et al. 2002), five 25 g raisin samples were taken from each of the processed, semiprocessed, and unprocessed raisin samples. Each sample was blended in 225 mL of PB for 2 min (Oster® blender). Samples were serially diluted in PB and planted on CC Petrifilm per the manufacturer's direction (3M Microbiology Products 1999). The CC Petrifilm was incubated at 35°C for 24 h, and the average CFU/g of total coliforms was calculated. The samples were also spread-plated on MacConkey Agar (DB, Sparks, MD) to confirm the CC petrifilm results. The MacConkey Agar plates were incubated at 35°C for 24 h for the enumeration of total coliforms.

## Results and Discussion

**Figure 1:**
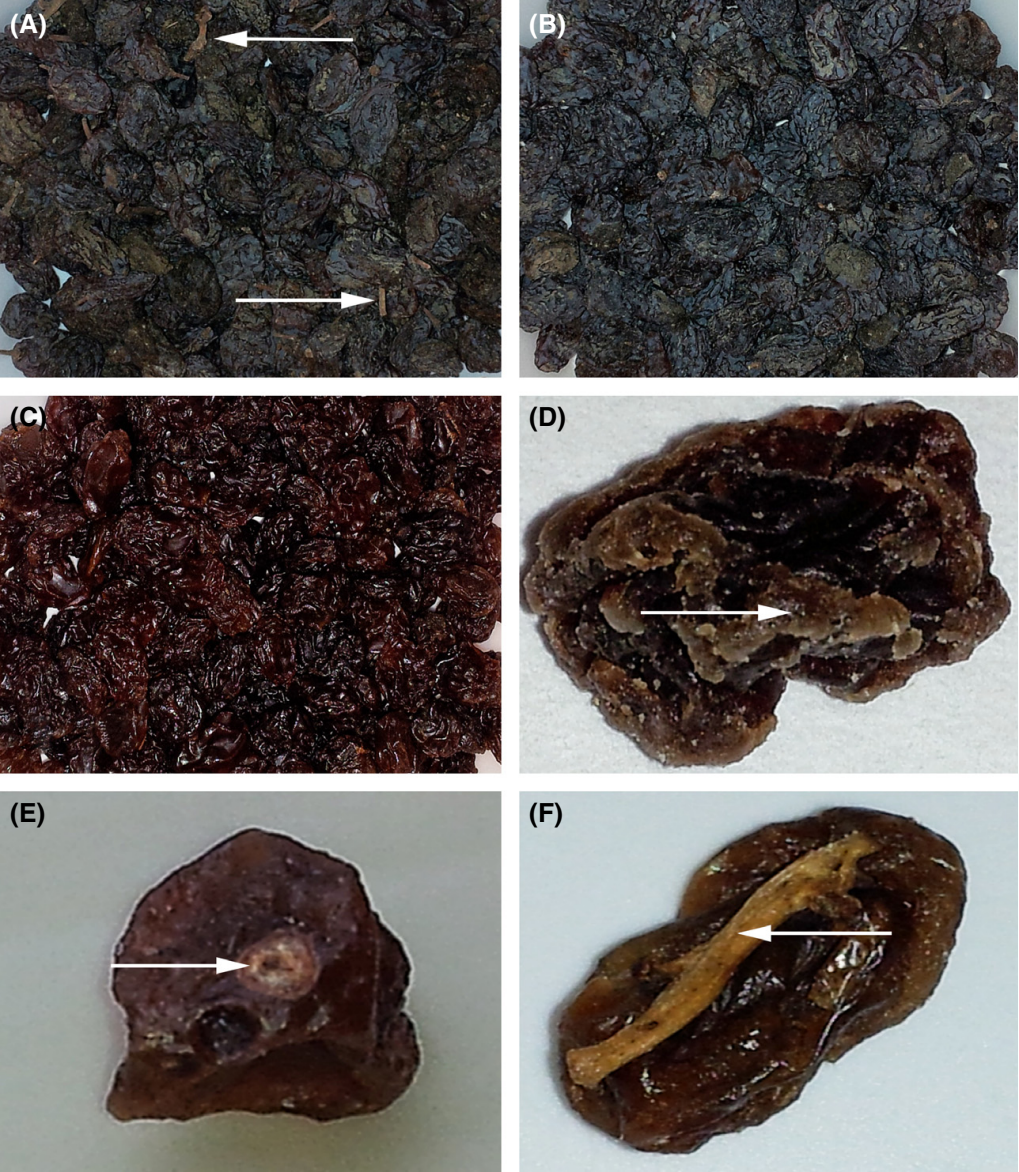
Images of representative Afghan raisin samples at different stages of processing where panel (A) is unprocessed (arrows show stem pieces); (B) is semiprocessed; and (C) is fully processed. Panel (D) is representative of a crystallized raisin (arrow shows area of crystallization). Panel (E) is characteristic of a damaged raisin (arrow shows damage). Panel (F) is an example of a raisin with an embedded stem (arrow shows stem).

In this study, the quality and safety aspects were evaluated for pre- (Fig.1A), semi- (Fig.1B), and postprocessed (Fig.1C) Afghan raisins from multiple production lots from a major raisin processor in Afghanistan. The goal of this study was to determine how Afghan raisins compared to safety and quality standards for U.S. raisins in order to determine feasibility of, and obstacles to, exporting Afghan raisins to the U.S. There are no other studies, to our knowledge, that exists for Afghan raisin quality and safety. Therefore, comparisons to other raisins grown in other countries and/or similar varieties were made for this study for quality assessment. The U.S. standards are similar to the Food and Agriculture Organization's *Codex Alimentarius* standards United Nations Food and Agriculture Organization (1981)used by the European Union and with which the Afghan government claims to be compliant (Bunnel and Safi 2013).

### Size and weight of Afghan raisin samples varied

Five of the processed raisin samples were tested, and all but one were found to be of “mixed” size. The remaining sample was “small”. As a part of the size consideration, no raisin sample should have more than 2–5% of the raisins being of “substandard development.” However, raisin maturity could not be tested as experienced USDA inspectors visually grade this attribute at import.

For comparison to a previous study (Kasimatis et al. 1979), the processed raisins were weighed and counted from each size class and the mean raisin weights were obtained. The Afghan raisins were somewhat lighter compared to what was reported for the Thompson Seedless raisins grown in California by Kasimatis et al. (1979) (Table1). The Afghan raisins weighed 0.32/0.25/0.19 g for raisins passing through 24/64 in. 22/64 in. and 20/64 in. perforations, respectively. The comparison values reported by Kasimatis et al. (1979) were 0.34/0.27/0.15 g, respectively.

**Table 1:** Summary of recommended values for select raisin attributes

Attribute measurement	Recommended values	Processed Afghan raisin values	US grade B or better?	Acceptable for import into US?
Embedded sand^1^	≤40 mg (per 100 g)	580 mg	NA^6^	No
Crystallization^2^	≤10% by weight	1.9%	Yes	Yes
Cap-stems^2^	≤25 cap-stems (per 454 g)	10.2 cap-stems	Yes	Yes
Pieces of stem^2^	≤2 stem (per 2.72 kg)	1.9 stems	Yes	Yes
Damage^2^	≤3% by weight	2.8%	Yes	Yes
Mold^2^	≤3% by weight	0.6%	Yes	Yes
Discoloration^2^	≤6% by weight	0.3%	Yes	Yes
Combined average damage mold and discoloration^2^	≤6% by weight	3.7%	Yes	Yes
Water Activity^2^	≤0.62 *a*_w_	0.52 *a*_w_	NA	Yes
Soluble solids^3^	≥19° Brix	17.16° Brix	NA	Unknown^7^
Titratable acidity^4^	≤2.3% dry weight	4.0%	NA	Unknown^7^
Weight^5^	≥0.25 g (per raisin)	0.28 g	NA	Yes
Size^2^	Select, small, or mixed	Small or mixed	NA	Unknown^8^

1 United Nations Food and Agriculture Organization (2013).

2 U.S. Department of Agriculture (1978).

3 Christensen (2000) and California Raisin Marketing Board (2014).

4 Christensen et al. (1995).

5 Kasimatis et al. (1977).

6 Does not apply to measuring US quality grade.

7 There is a maturity requirement in the 1978 USDA standard (and TSS/TA measure maturity), but the inspection protocol for imported raisins is visual. These raisins weren't mature compared to literature values but would likely pass a visual inspection if adequately hydrated.

8 The amount of raisins that were “substandard” could not be measured due to restricted sample availability; if ≥5% of the sample had been substandard, it would have been rejected.

To compare the weight of the Afghan raisins to the California Thompson Seedless grown raisins in the Kasimatis et al. (1977), study the moisture content would have to be adjusted to a common 15% moisture. When measuring raisin moisture content at room temperature (∼25°C), the water activity is 0.51–0.56 *a*_w_ at 13–15% moisture, and 0.55–0.62 *a*_w_ at 15–18% moisture (California Raisin Marketing Board 2014). The fully processed raisins averaged 0.52 *a*_w_, corresponding to a 13–15% moisture content range (Table1). The average weight of the Afghan raisins was 0.28 g across five processed raisin samples. The sample mean in the Kasimatis et al. (1977) study was 0.28 g; B and better raisins averaged 0.33 g, while C and substandard averaged 0.25 g and 0.12 g, respectively (Kasimatis et al. 1977). Given the processed raisins have approximately 13% moisture, when adjusted, the Afghan raisins would be somewhat heavier than the 0.28 g observed.

### Embedded sand and pieces of stem did not meet U.S. standards and industry practice, while cap-stems were acceptable

Three samples had a residual residue from the boiling process in addition to the sand/soil that added weight and gave inaccurate readings of sand/soil content. These samples were excluded when reporting the results (United Nations Food and Agriculture Organization 2013). The average sand/soil content in a 100 g raisin sample should not exceed 40 mg (Foreign Material Manual 2013). The Afghan samples exceeded this limit, with the average from two samples of fully processed raisins being 580 mg (Table1). Thus, the sample would fail import inspections. A 96 oz (2.72 kg) sample of raisins cannot contain more than 1 stem to achieve a grade A rating (U.S. Department of Agriculture 1978); industry practice is to follow the grade A threshold (Sun-Maid 2009; National Raisin Company 2010; Lion Raisins 2014). A grade B rating was assigned to the Afghan raisins, as they averaged 1.9 pieces of stem across 5 (2 kg) samples of fully processed raisins. A 16 oz sample of raisins must have fewer than 15 cap-stems to achieve a grade A rating (Fig.1F; U.S. Department of Agriculture 1978). The Afghan raisins sample was within this limit, averaging only 10.2 cap-stems across 5 (2 kg) samples of fully processed raisins.

### While percentage of damaged raisins exceeded U.S. standards, crystallization levels and color of the Afghan raisins met or exceeded the U.S. Grade A standard

A raisin sample cannot contain more than 2% by weight damaged raisins to achieve a grade A rating (U.S. Department of Agriculture 1978). Damaged raisins are defined as “raisins affected by sunburn, scars, insect injury, mechanical injury, or other similar means which seriously affect the appearance, edibility, storage quality, or shipping quality of the raisins” (U.S. Department of Agriculture 1978). Raisin damage occurs in fields and at packers throughout processing. Both the U.S. and EU have standards that specify maximum percent allowances of damaged raisins in an inspection sample. Acceptable maximum ranges are 4–9% (U.S. Department of Agriculture 1978; United Nations Food and Agriculture Organization 1981). Afghan raisins previously had lower damage counts than raisins from Australia, Greece, Turkey, and the USSR (Bongers et al. 1991), possibly due to the lack of mechanical harvesting. The processed Afghan raisins evaluated in this study were assigned a grade B rating as they had 2.8% by weight of damaged raisins averaged across 5 processed raisin samples (Fig.1E; Table1).

A raisin sample cannot contain more than 5% by weight crystallized raisins (U.S. Department of Agriculture 1978) to achieve a rating of grade A. The Afghan samples fell below this limit, with the average from 5 samples of fully processed raisins being 1.9% (Table1; Fig.1D). A raisin sample cannot contain more than 4% by weight discolored, damaged, or moldy raisins to achieve a grade A rating (U.S. Department of Agriculture 1978). There was an average of 0.3% discolored raisins in this study (Table1). The Afghan raisins met the standard, with a combined average of 3.7% discolored, damaged, or moldy raisins across 5 processed raisin samples (Table1). When combining the *L C h°* values for lightness (*L)*, color (*C*), and hue (*h*), respectively, the Afghan raisins appear to be a slightly lighter brown shade than the combined values from a 2007 study (Angulo et al. 2007) that quantified the color of Fiesta and Selma Pete raisin varieties. Although different varieties of raisins were used in the Angulo et al. (2007), study this was the best recent study that provided basic colorimeter measurements for raisins that did not receive special drying or pretreatments. The average lightness of unprocessed Afghan raisins was 28 *L*, with the raisins becoming lighter with each processing stage. The processed raisins had a lightness value of 20 *L*; this is lower than the 31–35 *L* found in the 2007 study (Angulo et al.). Afghan raisins had higher chroma values than the 2007 study (Angulo et al.); fully processed Afghan raisins averaged 7.6 *C* compared to 3.2–5.3 *C*. This indicates the Afghan raisins are more vividly colored. The Afghan raisins had hue angles of 54 *°h* for unprocessed raisins, shifting to become less yellow and more red with processing; average processed raisin values were 43 *°h*. The raisins analyzed in the 2007 (Angulo et al.) were more red-yellow than Afghan raisins, with values ranging 33–45 *°h*.

### While the moisture content was acceptable, TSS, and TA were below and above the optimal values

There is no U.S. grade associated with moisture content, but fully processed raisins should not exceed 18% moisture (U.S. Department of Agriculture 1978). The typical moisture content for raisins before processing is ∼10–16% (Christensen 2000). As described above, raisin moisture content at room temperature (∼25°C) corresponds to a water activity of 0.51–0.56 *a*_w_ at 13-15% moisture, and 0.55–0.62 *a*_w_ at 15-18% moisture (California Raisin Marketing Board 2014). Unprocessed Afghan raisins averaged 0.43 *a*_w_ and semiprocessed raisins averaged 0.46 *a*_w_. These low levels would inhibit microbial growth. The fully processed raisins averaged 0.52 *a*_w_, corresponding to the 13–15% moisture content range (Table1).

The primary measure of grape maturity is the Total Soluble Solid (TSS) content, as measured in degrees of Brix. The optimal minimum value for TSS for Thompson Seedless grapes, measured before drying into raisins, is above 19° Brix (Christensen 2000). Across nine samples of processed Afghan raisins, the average value of 17.16° Brix fell below the optimal value indicating that the grapes were harvested prematurely (Table1). Another measure of maturity is titratable acidity (TA), a more common measurement when determining maturity in raisins (as opposed to grapes). The optimal TA value for Thompson Seedless raisins is below 2.3% of the sample's dry weight (Christensen et al. 1995). The Afghan raisins were above this threshold, averaging 4.0% across all nine samples of processed raisins (Table1). The lower than optimal maturity has yield implications, as the drying ratio of grapes at 17° Brix is 4.87:1 compared to 4.39:1 at the recommended 19° Brix. Thus, raisins produced from the minimum recommended maturity yield 0.20 tons more from 9 tons of fresh grapes (Christensen 2000).

### Processing reduces yeasts, molds, and coliforms in Afghan raisins

There was an average of 3.0 × 10^4^, 1.3 × 10^5^, and 9.6 × 10^3^ CFU/g for the total aerobic plate count from each of the unprocessed, semiprocessed, and processed raisin samples, respectively (Table2). Most manufacturers limit total aerobic plate count values to less than an average of 2.0 × 10^4^ CFU/g (Sun-Maid 2009). Therefore, the unprocessed and semiprocessed raisins would not be acceptable. However, through the processing done by the manufacturer, the finished processed samples would be in the acceptable range for total aerobic plate count. The processed raisin samples had an average of 2.0 × 10^1^ CFU/g from PDA medium and 4 × 10^1^ CFU/g from CM medium for total yeasts (Table2). There was no yeast growth detected on the RB plates. The semiprocessed raisin samples had an average of 1.2 × 10^4^ CFU/g from PDA plates, 6.2 × 10^3^ CFU/g from RB plates, and 3.7 × 10^3^ CFU/g from CM plates (Table2). In addition, the unprocessed raisin samples had an average of 1.1 × 10^4^ CFU/g from PDA plates, 5.2 × 10^3^ CFU/g from RB plates, and 3.0 × 10^3^ CFU/g CM plates, respectively (Table2). The maximum allowable limit of yeasts for most manufacturers is 1.0 × 10^4^ CFU/g (Sun-Maid 2009). Therefore, the unprocessed and semiprocessed raisins were not within the acceptable average levels for yeasts. However, following processing done by the manufacturer, the finished processed samples would be in the acceptable range for total yeasts.

**Table 2:** Microbial growth in different media from the Afghanistan raisin samples

Media	Unprocessed^1^	Semiprocessed^2^	Processed^3^
Total plate count	3.0 × 10^4^	1.3 × 10^5^	9.6 × 10^3^
Potato dextrose agar			
Yeast	1.1 × 10^4^	1.2 × 10^4^	2.0 × 10^1^
Mold	5.8 × 10^5^	1.5 × 10^5^	5.2 × 10^3^
Rose-Bengal Agar			
Yeast	5.2 × 10^3^	6.2 × 10^3^	0
Mold	9.2 × 10^3^	1.2 × 10^4^	1.2 × 10^3^
Corn Meal Agar			
Yeast	3.0 × 10^3^	3.7 × 10^3^	4.0 × 10^1^
Mold	4.9 × 10^5^	1.1 × 10^5^	3.7 × 10^3^
MacConkey Agar	1.7 × 10^2^	3.3 × 10^2^	0
3M Petrifilm™ Coliform	4.8 × 10^3^	9.9 × 10^3^	0

1 CFU/g in based on 25 g samples of unprocessed raisins.

2 CFU/g in based on 25 g samples of semiprocessed raisins.

3 CFU/g in based on 25 g samples of fully processed raisins.

Mold growth was detected on all PDA, RB, and CM plates. Processed raisin samples had an average of 5.2 × 10^3^ CFU/g from PDA plates, 1.2 × 10^2^ CFU/g from RB plates, and 3.7 × 10^3^ CFU/g from CM plates. There was more mold growth in the semiprocessed and unprocessed raisin samples compared to the processed raisin samples. The semiprocessed raisin samples had an average of 1.5 × 10^5^ CFU/g from PDA plates, 1.2 × 10^4^ CFU/g from RB plates, and 1.1 × 10^5^ CFU/g CM plates, respectively. Unprocessed raisin samples had an average of 5.8 × 10^5^ CFU/g from PDA plates, 9.2 × 10^3^ CFU/g from RB plates, and 4.9 × 10^5^ CFU/g from CM plates (Table2). Most raisin manufacturers limit the total mold values to less than an average of 1.0 × 10^4^ CFU/g (Sun-Maid 2009). Therefore, the unprocessed and semiprocessed raisins would not be acceptable. However, following processing done by the manufacturer, the finished processed samples would be in the acceptable range for molds.

As an additional test of food safety, total coliforms were enumerated from the Afghan raisin samples using CC 3M Petrifilm™ and MacConkey agar medium. Coliforms are not usually regarded as pathogens, but testing for them has been the traditional hygienic quality indicator for food production conditions. Processing reduced the total coliforms from an average of 4.8 × 10^3^ CFU/g to zero using CC petrifilm, while the semiprocessed raisin sample had 9.9 × 10^3^ CFU/g total coliforms. The enumeration of total coliforms using MacConkey medium from the processed raisin samples also had no growth of coliforms. Raisin manufactures require fewer than 10 CFU/g of total coliforms in the final product, so that the processed samples would be acceptable, but the semiprocessed and unprocessed raisin samples with an average of 3.3 × 10^2^ CFU/g and average 1.7 × 10^2^ CFU/g total coliforms, respectively, would not (Sun-Maid 2009; Lion Raisins 2014). The processing used by the manufacturer was successful in reducing the number of total coliforms associated with the raisin samples.

### Potential implications of meeting or failure to meet quality and safety standards on Afghan raisin exports

The real raisin prices in 2011 dollars have risen steadily since 2001, and the total quantities of raisins traded have increased steadily over the past 50 years (United Nations Food and Agriculture Organization 2013). The increased quantity and price of raisins on the global market since 2011 implies there is unmet demand for raisins on which Afghanistan may be able to capitalize. Afghanistan is estimated to produce 33,000 MT of raisins in MY 2013/14 (Bunnel and Safi 2013), which is 2.75% of the 1.2 million MT global supply (Halstead 2013). Russia is currently the largest importer of Afghan raisins. Their raisin imports from Afghanistan were 13,525 MT and accounted for 29% of all Russian raisin imports in 2011 (United Nations Food and Agriculture Organization 2013). The Afghan government, the USDA, and Afghan raisin packers would like to increase the export of Afghan raisins to the U.S. and EU, where there is a market for higher-quality raisins (Chehrezad and Fite 2005). However, the quality of Afghan raisins and the potential price premium for improved raisin quality that meets U.S./EU standards are unknown. The deficiencies noted above (embedded sand, stem pieces, and maturity) are influenced by growers, whereas washing processes at the packer reduce embedded sand and stem pieces. The lack of influence the packers have on maturity deficiencies in raisins makes quality improvement challenging, as the packers currently procure from commission agents and have little direct communication with growers.

Afghan raisin packers interested in exporting their product to countries with high food quality and safety standards should focus on reducing embedded sand and stem pieces, and procuring more mature raisins. Although this study did not find raisin crystallization to be a problem and did not test for pesticide residues, the FDA has rejected Afghan raisin shipments in the past four years for these issues as well (Osmani, 2013, email comm.; U.S. Department of Health and Human Services 2014). To mitigate maturity and pesticide residue problems, the packers will need to exert more control over their raw products, potentially by incentivizing growers. In order for packers to encourage intermediaries to source higher maturity raisins, all parties will need to better understand why Afghan growers harvest raisin grapes earlier than recommended in the viticulture literature. Such a study could also explore the feasibility of growers harvesting grapes destined for *aftabi* raisins later than grapes destined for fresh consumption. For example, growers may harvest all of their grapes for table grape sales, and process grapes they could not sell into the fresh market into raisins. Labor availability might also be a factor influencing harvest practices. More insight into production/harvesting technology and harvesting decisions could help develop management plans to harvest grapes for table grape sales based on marketing estimates. To mitigate crystallization issues, the packer likely needs to improve shipping conditions (temperature and humidity) and reduce time from initial processing to final import inspection. The packers' own processes may be able to reduce embedded sand and stem pieces to acceptable levels.

## Conclusion

Future studies should focus on gaining a better understanding of (i) what Afghan growers can feasibly do to control or improve the factors of production to make sun-dried raisins more acceptable to U.S. and EU markets; (ii) the costs for packers to increase quality; and (iii) the potential financial benefit to packers from improved raisin quality, as well as what incentives packers can then provide to growers for improved quality. Understanding these dynamics will aid in the improvement of the quality attributes of Afghan raisin to help facilitate export to other markets.
